# Implementing and evaluating a fully functional AI-enabled model for chronic eye disease screening in a real clinical environment

**DOI:** 10.1186/s12886-024-03306-y

**Published:** 2024-02-01

**Authors:** Christos Skevas, Nicolás Pérez de Olaguer, Albert Lleó, David Thiwa, Ulrike Schroeter, Inês Valente Lopes, Luca Mautone, Stephan J. Linke, Martin Stephan Spitzer, Daniel Yap, Di Xiao

**Affiliations:** 1 Department of Ophthalmology, University Medical Center Hamburg - Eppendorf, Martinistr. 52, 20249 Hamburg, Germany; 2 TeleMedC GmbH, Raboisen 32, 20095 Hamburg, Germany; 3 Department of Otorhinolaryngology, University Medical Center Hamburg - Eppendorf, Martinistr. 52, 20249 Hamburg, Germany; 4 Zentrum Sehestaerke, Martinistraße 64, 20251 Hamburg, Germany; 5 TeleMedC Pty Ltd, 61 Ubi Avenue 1, #06-11 UBPoint, Singapore, 40894 Singapore; 6 TeleMedC Pty Ltd, Brisbane Technology Park, Level 2, 1 Westlink Court, Darra, QLD 4076 Australia

**Keywords:** Artificial intelligence, Telemedicine, AMD, Glaucoma, Diabetic retinopathy, Screening, Digital color fundus imaging

## Abstract

**Background:**

Artificial intelligence (AI) has the potential to increase the affordability and accessibility of eye disease screening, especially with the recent approval of AI-based diabetic retinopathy (DR) screening programs in several countries.

**Methods:**

This study investigated the performance, feasibility, and user experience of a seamless hardware and software solution for screening chronic eye diseases in a real-world clinical environment in Germany. The solution integrated AI grading for DR, age-related macular degeneration (AMD), and glaucoma, along with specialist auditing and patient referral decision. The study comprised several components: (1) evaluating the entire system solution from recruitment to eye image capture and AI grading for DR, AMD, and glaucoma; (2) comparing specialist’s grading results with AI grading results; (3) gathering user feedback on the solution.

**Results:**

A total of 231 patients were recruited, and their consent forms were obtained. The sensitivity, specificity, and area under the curve for DR grading were 100.00%, 80.10%, and 90.00%, respectively. For AMD grading, the values were 90.91%, 78.79%, and 85.00%, and for glaucoma grading, the values were 93.26%, 76.76%, and 85.00%. The analysis of all false positive cases across the three diseases and their comparison with the final referral decisions revealed that only 17 patients were falsely referred among the 231 patients. The efficacy analysis of the system demonstrated the effectiveness of the AI grading process in the study’s testing environment. Clinical staff involved in using the system provided positive feedback on the disease screening process, particularly praising the seamless workflow from patient registration to image transmission and obtaining the final result. Results from a questionnaire completed by 12 participants indicated that most found the system easy, quick, and highly satisfactory. The study also revealed room for improvement in the AMD model, suggesting the need to enhance its training data. Furthermore, the performance of the glaucoma model grading could be improved by incorporating additional measures such as intraocular pressure.

**Conclusions:**

The implementation of the AI-based approach for screening three chronic eye diseases proved effective in real-world settings, earning positive feedback on the usability of the integrated platform from both the screening staff and auditors. The auditing function has proven valuable for obtaining efficient second opinions from experts, pointing to its potential for enhancing remote screening capabilities.

**Trial registration:**

Institutional Review Board of the Hamburg Medical Chamber (*Ethik-Kommission der Ärztekammer Hamburg*): 2021-10574-BO-ff.

## Background

As the global prevalence of chronic eye diseases such as diabetic retinopathy (DR), glaucoma, and age-related macular degeneration (AMD) continues to rise, early detection and management of these conditions are increasingly critical. Traditionally, the screening and diagnosis of these diseases have relied heavily on manual inspection and interpretation of retinal images by ophthalmologists. However, this process is not only time-consuming and resource-intensive but also susceptible to inter-observer variability and human error.

In recent years, artificial intelligence (AI), and more specifically deep learning, has emerged as a powerful tool to revolutionize the field of ophthalmology. AI algorithms have demonstrated high performance in the automated grading the severity of DR, AMD, and glaucoma using retinal images. These advancements have not only shown the potential to enhance diagnostic accuracy and efficiency but also to reduce the burden on healthcare systems and improve patient outcomes.

Recent advances in AI have revolutionized the field of DR grading using retinal images. Early publications in 2016 by Gulshan et al. showed the effectiveness of a deep learning algorithm for detecting referable DR from colour fundus photographs with high sensitivity and specificity. Subsequent studies by Abràmoff et al. (2018), Ting et al. (2017), and Gargeya and Leng (2017) demonstrated similar performance [1–4]. In a recent study, Li et al. (2022) developed a deep ensemble algorithm capable of detecting both diabetic retinopathy (DR) and diabetic macular edema (DME) [5], which exhibited performance that was comparable to or even surpassed that of ophthalmologists. Several recent review papers by Sebastian (2023), Tsiknakis (2021), and Dubey (2023) offer comprehensive insights into this rapidly evolving area and can be referenced for further exploration [6–8].

AI has also shown promising results in detecting and classifying AMD severity from retinal images. Burlina et al. (2018), Ting et al. (2017), and Peng et al. (2019) showed that deep-learning models could achieve higher accuracy in the automated classification of patient-based AMD severity using bilateral colour fundus photographs and outperformed retinal specialists [3, 9, 10]. Several recent review papers in this area can be referenced, including those by Leng et al. (2023), Paul et al. (2022), and Dong et al. (2021) on AI for AMD screening using colour fundus images or OCT images [11–13].

For glaucoma detection, the early AI models were primarily focused on analysing various features such as the optic disc and cup-to-disc ratio [14, 15]. Further studies have explored the use of retinal vessel segmentation and texture analysis to improve the performance of AI-based glaucoma detection systems [16, 17]. In a systematic review and meta-analysis conducted by Buisson et al. (2021), deep learning models demonstrated similar performance to ophthalmologists in diagnosing glaucoma from fundus examinations [18]. The reviews (Atalie 2020, Yousefi 2023) discuss its potential to improve diagnostic capabilities but also point out its challenges and the need for careful validation in clinical practice [19, 20].

Despite the promising research and developments, their translation into real-world clinical practice remains a challenging endeavour. To date, there has been a gradual deployment of AI models in software systems for DR screening over the past six years. Several AI-based screening systems, such as IDx-DR, Thirona Retina, Retmarker, EyeArt, iGradingM, Eyetelligence Assure, Retinalyze, TeleEye MD, Airdoc-AIFUNDUS, and SELENA+, have been published and deployed in various clinical settings.

IDx-DR was validated on 900 patients in primary care sites in the USA, achieving a sensitivity of 87.2% and a specificity of 90.7% on the 819 participants that were analysable [1]. Based on these results, it gained the FDA certificate for use by healthcare providers for automatically detecting more than mild diabetic retinopathy (mtmDR). IDX-DR was also validated in the Hoorn Diabetes Care System in the Netherlands, achieving a sensitivity of 91% and a specificity of 84% for detecting referable DR [21]. In a pilot study, IDx-DR showed a higher percentage agreement with human ophthalmologists in both DR positive and DR negative cases, suggesting it may be more reliable for autonomous screening [22].

EyeArt was validated in more than 30,000 patients in the English Diabetic Eye Screening Programme in the UK, achieving a sensitivity of 95.7% and a specificity of 54.0% for referable retinopathy [23]. In another validation in the USA,it achieved similar sensitivities and specificities for detecting both mtmDR (95.5% sensitivity and 85.3% specificity) and vision-threatening diabetic retinopathy (VTDR, 95.2% sensitivity and 89.5% specificity) on 893 patients [24]. SELENA + was validated on 1574 patients in a mobile screening program in Zambia, achieving a sensitivity of 92.25% and specificity of 89.04% for referable DR and a sensitivity of 99.42% for VTDR and a sensitivity of 97.19% for DME [25]. In a small-cohort pilot study on tele-ophthalmology, it demonstrated 100% referral accuracy for known 9 diabetic retinopathy patients among 69 validation patients [26]. An offline AI-based DR grading system was validated on populations (236 participants) from two endocrinology outpatients and three Aboriginal Medical Service clinics in Australia, achieving a sensitivity of 96.9% and a specificity of 87.7% for detecting referable DR [27]. The VoxelCloud Retina was validated on 15,805 patients at 155 diabetes centres in China, achieving an 83.3% sensitivity and a 92.5% specificity to detect referable DR [28]. In a recent study, an AI system RAIDS that can detect seven eye conditions, including common abnormalities like DR, ARMD, and glaucoma, was validated in real-world clinical settings [29]. The system achieved sensitivities and specificities for DR, ARMD, and referrable glaucoma of 83.7% and 88.1%, 81.3% and 98.6%, and 97.6% and 95.0%, respectively.

While the abovementioned systems have been validated and approved for clinical use, there is limited information available regarding their real-world performance and acceptance among end-users, indicating a need for further research in this area. Only the study of the offline AI-based DR grading system [27] investigated the experience and acceptance, which is the first for an AI system to complete the accuracy analysis and the system’s end-user experience analysis. Conversations regarding the influence of socio-environmental factors on deep learning model performance have been relatively scarce. A noteworthy contribution to this discourse comes from Beede et al. at Google Health who conducted a human-centered observational study on a deep learning system in clinical care in Thailand [30]. Their research highlighted the impact of end-users and environmental factors, including lighting conditions, patient expenses, and model threshold settings, etc. It underscores the urgency to develop methodologies for designing and evaluating deep learning systems in clinical settings, emphasizing collaboration with the Human-Computer Interaction (HCI) community [31, 32].

To address these gaps, this study aims to implement and evaluate a fully-integrated hardware and software solution and an automatic workflow in a real clinical environment. By evaluating the system’s performance, diagnostic performance, and user experience, we hope to shed light on the feasibility, acceptability, and accuracy of AI-assisted chronic eye disease screening. Furthermore, we aim to understand the challenges and opportunities associated with the real-world deployment of AI in ophthalmic disease screening and management. To the best of our knowledge, this study represents one of the pioneering attempts to incorporate screening for the three chronic diseases within an integrated retinal imaging and AI-grading system supported by a cloud solution.

## Methods

The study uses a cloud-based tele-ophthalmological platform (TeleEye MD) combined with a retinal camera (DRS Plus, Icare Finland Oy, Finland) and a data transmission device. The study focused on several studying points:

1. Workflow for chronic eye disease screening from patient retinal image capture till report generation;
2. AI-assisted grading for DR, AMD and glaucoma;
3. Human grader’s audit based on the AI grading results;
4. Feedback from patients and health professionals;
5. System efficacy.

### Participants

The patients included in this prospective study were recruited at the Ophthalmology Outpatient Department of medical retina and glaucoma, of the University Medical Center Hamburg-Eppendorf, Germany. The medical staff approached the patients and explained the goals of the study and the examinations that had to be performed. After informed consent was given by all patients, a trained study nurse (US) who was hired to support the study, performed patient examinations using the screening system. All patients recruited in the study completed the necessary examinations. The study patients shared the same premises and the same hardware as all other patients. The recruitment occurred from December 2021 to October 2022.

### Ethics and inclusion criteria

This prospective study was registered and approved by the Ethics Review Board of the Medical Association of Hamburg (process number: 2021-10574-BO-ff) and follows the recommendations of the Declaration of Helsinki. The patients could withdraw from the study at any time by informing the supervisors. Inclusion criteria were an age of at least 18 years and eyes in which clear media allowed a sharp fundus photo.

### Screening workflow

Patients underwent an ocular examination utilizing a digital colour fundus imaging device. The process was facilitated by a study nurse using a patient registration and data transmission tool - the bridging device. To maintain confidentiality, unique project IDs were assigned to each patient’s data, which were then transferred to the cloud-based system. This system, powered by an integrated AI, graded the patients’ colour fundus images for DR, AMD and glaucoma. These AI-graded images were subsequently managed by the cloud system and subjected to audit by the study’s specialist (CS).

The patients performed the following examinations: objective refraction, non-contact eye pressure measurement, best corrected visual acuity (BCVA) and after pupillary dilation, fundus photography of the retina (optic disc, macula and retinal periphery), and anterior chamber photos focused on the lens.

**Fig. 1:**
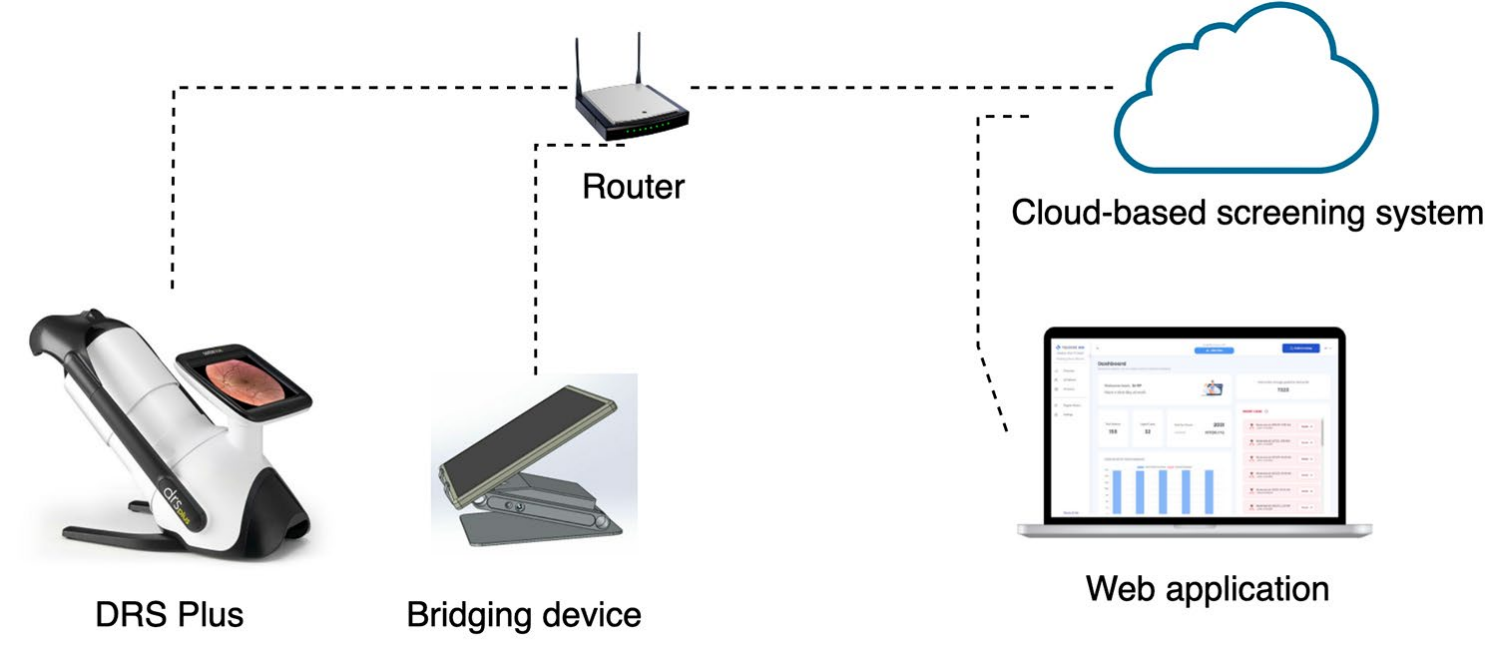
Hardware configuration for the screening service

The following picture (Fig. 1) illustrates the hardware and its configuration used in the workflow.

**Fig. 2:**
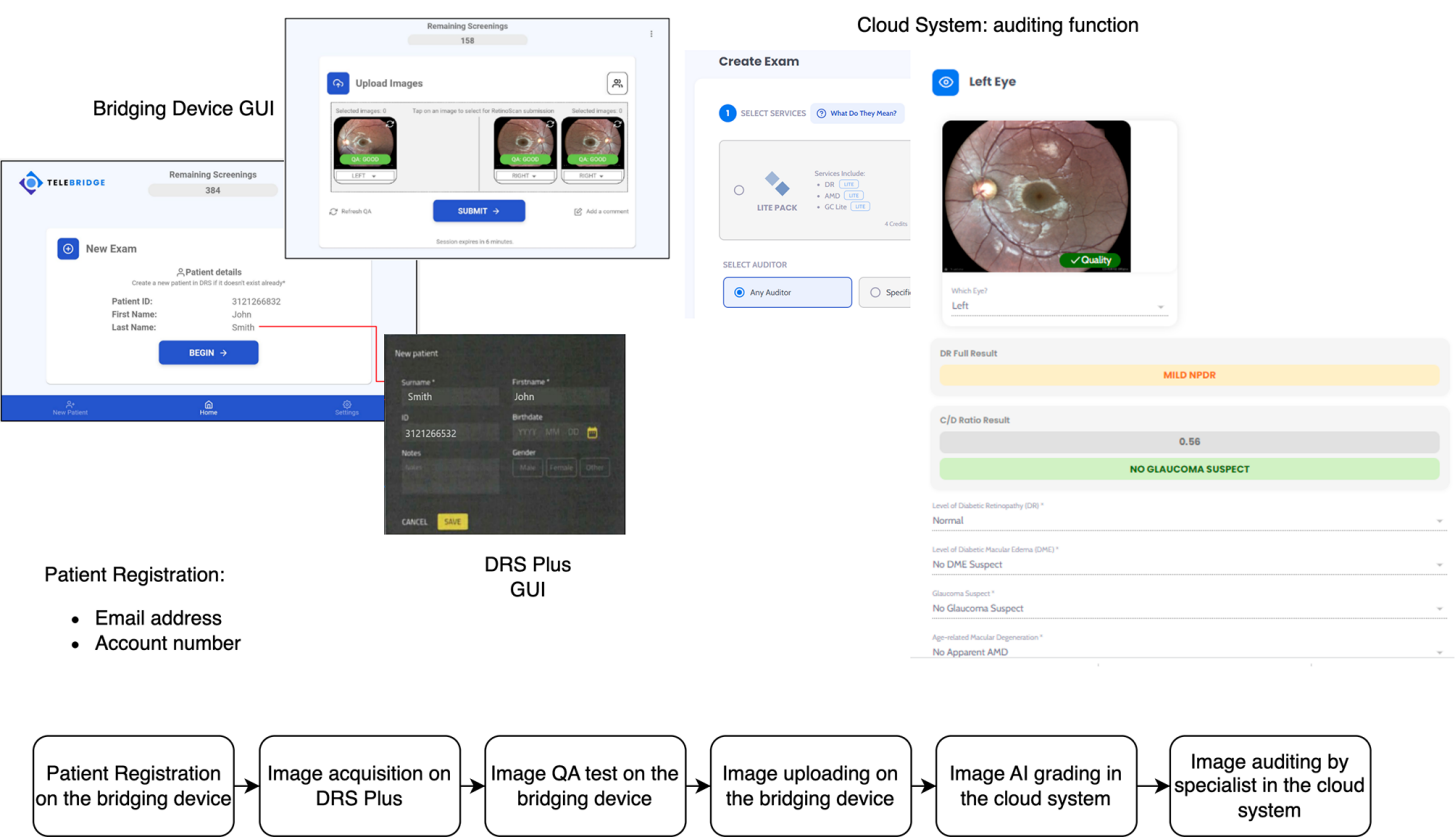
Screening workflow and software configuration

Figure 2 depicts the workflow of the screening process. It consists of the following steps:

1. The study nurse inputs the recruited patient’s information, including the patient’s project ID, ethnicity, age and gender, on the bridging device and transfers them into the cloud system.
2. The study nurse guides the patient into position for eye image capture using the DRS Plus camera. Once the patient is properly positioned, the study nurse activates the camera and captures an image of the patient’s retina. One macula-centered image (45-degree field of view) per eye is captured. The image is instantly transmitted to the bridging device. The bridging device uses its cloud service to analyze the image and provides a quality assessment (QA) score within seconds. If the QA score is “inadequate,” the bridging device will notify the study nurse on the screen, prompting them to recapture the image.
3. After the QA process, on the bridging device, the study nurse selects one or two macula-centered images from each eye and submits them to the patient’s cloud account for DR, AMD, and glaucoma gradings by the AI.
4. An auditor (CS) with an auditor account (i.e., an ophthalmologist), can log into the cloud system and view the patient’s colour fundus images from both eyes with their raw resolutions and select human grading options for DR, AMD, and glaucoma. Then, a final report can be generated.
5. The study nurse has access to the cloud web portal to check patiens’ report readiness. Once the final report is ready, the study nurse can download the report from the platform.

### Disease grading protocol

This study’s disease grading protocols were established on the foundations of the International Clinical Diabetic Retinopathy Disease Severity Scale and the International Clinical Diabetic Edema Disease Severity Scale, used for DR and DME respectively [33]. DR grading ranged from the grading levels ‘No Apparent Retinopathy’, ‘mild Non-Proliferative DR (NPDR)’, ‘moderate NPDR, severe NPDR’, and ‘proliferative DR’. DME grading was categorized as ‘Diabetic Macular Edema Absent’, progressing through ‘Mild’, ‘Moderate’, and ‘Severe’ DME. In the case of AMD, the grading commenced from ‘No Apparent AMD’, escalating to ‘Early’, ‘Intermediate’, and ‘Advanced’ AMD. Glaucoma grading was simplified to ‘Referable’ (suspect glaucoma) or ‘Non-Referable’ based solely on image analysis of the optic nerve head. No further analysis has been performed e.g. IOP (intraocular pressure) measurement or nerve fibre layer analysis. Lens opacity was evaluated as ‘Normal’, ‘Non-Significant Media Opacity’, and ‘Significant Media Opacity’. During the auditing process, the auditor could select the appropriate grading options for DR, AMD, and glaucoma levels, as well as lens opacity status, and the grading results would be reflected in the final report.

However, the AI grading process only provided referable or non-referable results for DR, AMD, and glaucoma, where “referable DR” indicated more than ‘Mild NPDR’, and “referable AMD” indicated any condition more severe than ‘Early AMD’. If the configuration of the optic nerve head was suspected, then referral to an ophthalmology specialist was advised. If the image is ungradable judged by the AI grading, the patient should be considered referred.

### Automatic AI grading algorithms and platform settings

The DR grading method was described in an early paper with subsequent improvements made afterwards [34]. The development of the AMD grading model was based on an EfficientNet deep learning backbone with customized classification layers. The model was trained and validated on 4,218 images and achieved a specificity of 95.23% and a sensitivity of 98.14%. The development of the glaucoma grading algorithm was based on EfficientNet deep learning backbone with customized classification layers. 3,2828 images were used for the deep learning model’s training and validation. The model achieved a sensitivity of 91.48% and a specificity of 92.94%.

The three AI models were integrated into the cloud system by utilising the Lambda Service approach provided by Amazon AWS. Besides the AI grading and human auditing, the cloud system also provides the functions of clinic management and patient health data management. System testing and staff training.

Prior to the patient recruitment process, the engineering team meticulously tested both the hardware configuration and the comprehensive screening workflow. Two demonstrations were conducted by the engineering team to the clinical staff in the study. The screening organization account and the user accounts for screening study nurses, managers and auditors were created and the hardware and software user manuals and training materials were provided. The training for the clinical staff was provided by the two trainers (NO & AL). The training consisted of how to use the bridging device for patient registration and how to use the DRS Plus camera for image capture, as well as checking image quality and exam submission for the imaging study nurse. The auditor (CK) was trained on how to log in and use the auditing page in the cloud system. Grading protocols were discussed and conformed to. Following the training, the staff’s utilization and proficiency with the system were overseen until they demonstrated independent capability in its operation.

The study nurse performing the examination was also trained to explain the project information sheet and patient consent form to recruited patients. Patient questionnaire forms were provided to patients to collect patient feedback for the AI screening service.

### Data analysis

#### System efficacy analysis

The efficacy of the AI-based eye disease screening system was analyzed by addressing several critical aspects. Firstly, real-time QA was evaluated during the image acquisition process. Secondly, the AI grading procedure was assessed. The images were analyzed using cloud computing to produce three different diseases’ grades, which were then shown on the bridging device. The promptness in the generation and availability of these results to the clinicians was analyzed. The final aspect evaluated was the time required for a human auditor to review a single case.

#### Disease grading analysis

All de-identified data were exported from the AWS cloud server and imported into an Excel spreadsheet for further analysis.

The exported data include three main parts: (1) general patient information recorded; (2) AI grading results: AI-based gradings for left/right eye identification, image QA, and AMD, DR, Glaucoma three diseases finally; (3) auditing results: auditor’s grading for DR levels, DME levels, AMD levels, glaucoma normal/suspect, image ungradable/gradable, and lens opacity, auditor comment, follow-up screening period, and ophthalmologist referral period.

Sensitivity, specificity, positive predictive value (PPV), negative predictive value (NPV) and AUC were calculated to evaluate the grading accuracy of the three diseases.

#### Participant feedback analysis

Following the acquisition of images and the initial grading by the AI, patients were invited to participate in a comprehensive survey aimed at capturing their experiences and attitudes towards the AI-based eye disease screening system. To gather this valuable feedback, a questionnaire consisting of four questions was administered (Table 1).

**Table 1:** Questions in the questionnaire form for participants

Questions	Selections*
How satisfied were you with your experience with the AI-based eye disease screening system? Please explain the reason you gave the score.	(1) Very dissatisfied; (2) Dissatisfied; (3) Neutral (4) Satisfied; (5) Very satisfied
Were you satisfied with the time consumption for completing your eye disease screening? Please explain the reason you gave the score.	(1) Very dissatisfied; (2) Dissatisfied; (3) Neutral (4) Satisfied; (5) Very satisfied
Did you use the web portal’s mobile app for your registration? If “Yes”, was it simple to use the app? Please explain the reason you gave the score. (note: this question is not applicable for the study)	(1) So difficult; (2) Difficult; (3) Neutral (4) Easy; (5) So easy
Considering your complete experience with our medical facility, how likely would you be to recommend us to a friend or colleague? Please explain the reason you gave the score.	(1) Not at all; (2) Not recommendable; (3) Neutral (4) Recommendable; (5) Very recommendable
Free comments and suggestions.	

*For each question, patients were instructed to select only one option from the five provided, with each option assigned a score ranging from 1 (lowest rating) to 5 (highest rating) based on the corresponding item number.

## Results

A total of 231 patients were recruited to the study with 125 being male and the remaining 106 being female. This gives a gender ratio of approximately 1.18 males to every female.

The age summary in the dataset showed a mean age of approximately 63 years old. The standard deviation is 16.9, indicating that the ages are fairly spread out. The youngest individual in the dataset is 19 years old, while the oldest is 95. The median age is 66, and the interquartile range (IQR) is from 54.5 to 76.0 years old. It is worth noting that all 231 patients in the dataset self-identified as Caucasian.

### Gradable patients and images

**Fig. 3:**
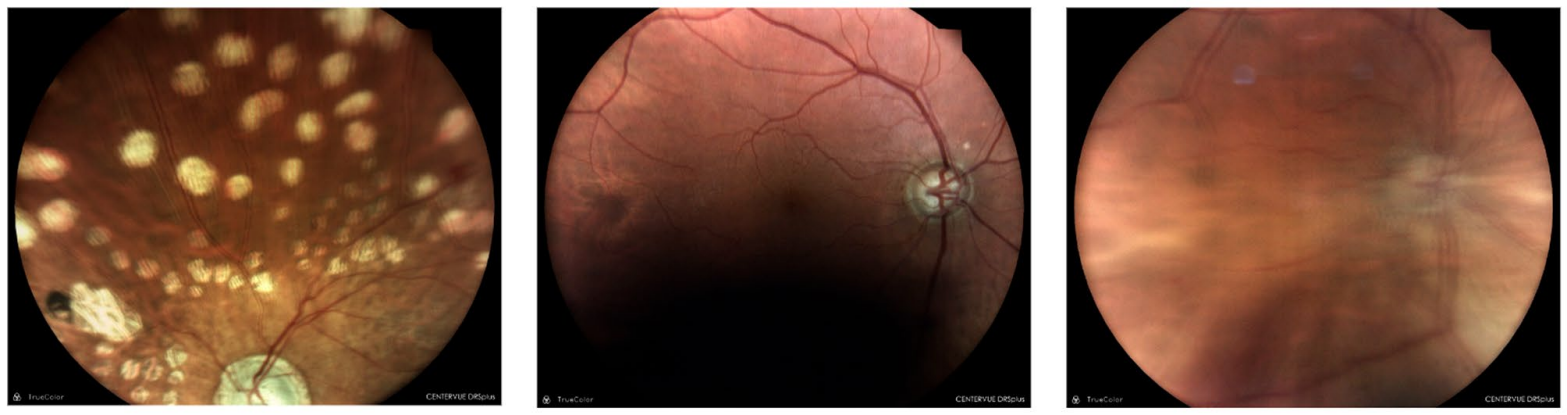
Examples of ungradable images

Out of the 231 patients initially assessed, four cases had both eyes ungradable according to the auditor, a finding which was agreed upon by the AI. Interestingly, in one patient, the AI determined both eyes to be ungradable, but this assessment was not shared by the auditor. Among the remaining patients, eight had a single ungradable eye according to the auditor, and of these, the AI agreed with six of the assessments. On the other hand, the AI determined that 11 patients had a single ungradable eye, but the grader disagreed with this assessment. These findings suggest that there may be discrepancies between the AI and human graders in assessing ungradable eyes in patients. The ungradable images include “image half blurry”, “image half dark” and “significant media opacity” etc. situations (Fig. 3).

### Disease distribution

In the auditing process, our online auditing page enabled the auditor to choose the ‘ungradable’ option for each of the three diseases independently.

Among the 231 patients assessed by the auditor, a total of 27 patients were identified as referable DR patients, 33 patients were identified as referable AMD patients, and 89 patients were classified as referable (suspect) glaucoma cases.

In terms of ophthalmologist referrals, as determined by the auditor, 65 patients only needed to undergo regular screening without referrals. However, 149 patients needed to be referred to ophthalmologists within 3 months, 12 patients required appointments within 4 weeks, and 5 patients needed urgent referrals within 1 week.

### AI grading performance

The performance of the three models for grading DR, AMD, and glaucoma diseases was compared with the auditor’s assessment results. In the context of disease screening and patient referral, the evaluation of the performance of the three models was conducted at the patient level. The initial step in the workflow involved left/right eye identification based on colour fundus images. Given that these images are macula-centred and readily distinguishable, the left/right eye identification model achieved 100% accuracy. When calculating sensitivity and specificity, any eyes deemed ‘ungradable’ by the auditor for a specific disease grading were categorized as ‘referable’ cases for that disease. Conversely, if an image or eye is assessed as ‘ungradable’ by the QA model but is, in fact, considered gradable and normal by a human, it is counted as a false positive case. It is important to note that the PPV and NPV can be influenced by the disease prevalence value used. Considering that the sensitivity values for all three disease gradings are above 90%, with only a few misclassified cases, the results are presented here in the three tables (Tables 2, 3 and 4). Table 5 presents the statistical data of the false positive classifications under each disease and their “ophthalmologist referral” decisions from the auditor’s assessment based on other abnormal findings. The data contribution to the s miss classifications and their final referral decisions will bediscussed in the discussion section.

**Table 2:** DR AI grading accuracy summary

Statistic	Value	95% CI
Sensitivity	100.00%	87.23–100.00%
Specificity	80.10%	73.98–85.32%
Positive Predictive Value *	37.33%	25.90–49.91%
Negative Predictive Value *	100.00%	97.79–100.00%
AUC	0.90	87–93%

* These values are dependent on German disease prevalence 10.60%

**Table 3:** AMD AI grading accuracy summary

Statistic	Value	95% CI
Sensitivity	90.91%	75.67–98.08%
Specificity	78.79%	72.43–84.26%
Positive Predictive Value *	29.26%	19.12–41.16%
Negative Predictive Value *	98.90%	95.78–99.89%
AUC	0.85	79–90%

* These values are dependent on German disease prevalence 8.80%

**Table 4:** Glaucoma AI grading accuracy summary

Statistic	Value	95% CI
Sensitivity	93.26%	85.90–97.49%
Specificity	76.76%	68.94–83.43%
Positive Predictive Value *	7.50%	3.43–13.89%
Negative Predictive Value *	99.82%	96.49–100.00%
AUC	0.85	81–89%

* These values are dependent on German disease prevalence 1.98%

**Table 5:** Ophthalmologist referral decided by the auditor for the patients under the false positive cases

Ophthalmologist Referral Decision from Auditor					
False Positive DR		False Positive AMD		False Positive Glaucoma	
Period	Number of Patients	Period	Number of Patients	Period	Number of Patients
Within 1 week	0	Within 1 week	1	Within 1 week	1
Within 4 weeks	4	Within 4 weeks	3	Within 4 weeks	3
Within 3 months	33	Within 3 months	33	Within 3 months	17
Not Required	4	Not Required	5	Not Required	12

### System efficacy

Table 6 presents the performance metrics of four independent AWS Lambda services used in this study. The QA Lambda service is specifically employed during the image quality assessment step, while the remaining three Lambda services are concurrently triggered during the image grading step, enabling simultaneous execution. The average process time, measured after the services’ warm-up periods, represents the duration required for each Lambda service to complete its execution. Additionally, the memory allocation for each service is determined based on the structural characteristics of the deep learning models employed.

**Table 6:** Processing time of the deep learning models in the AWS cloud

AWS Lambda Service	Process Time (ms)*	Memory allocation (MB)
QA	1000	1536
DR grading	2000	1536
AMD grading	1500	1024
Glaucoma grading	4000	4096

*Average process time of the warmed-up lambda services

Based on the user observations and experiences, several other important parameters reflect the efficacy of the system too:

- Registering a patient takes an average of 45 s.
- After capturing an image, the QA result is typically available in 6 s for it.
- When submitting two images to obtain results, the process takes an average of 46 s for uploading and grading the images. Alternatively, grading images only takes an average of 30 s.
- Auditing time for one case by the auditor is less than 5 min.

### Participant feedback

After completing the examinations for the study, patients were kindly asked to give us feedback by completing a questionnaire. A total of 12 questionnaire forms were collected from the participants to assess their feedback on the screening system. Regarding the question on “satisfaction with the screening system,” the average rating score was 4.00 on the rating scale from 1 (lowest) to 5 (highest). Out of the 12 patients, 4 selected “neutral,” 4 selected “satisfied,” and 4 selected “very satisfied.” Among the seven comments received for this question, five patients expressed that the system was “Easy and quick” or simply “Easy.” One patient mentioned discomfort, stating that the device was “too tight in the mouth plus nose area,” which was related to the camera usage. Another patient mentioned having “no knowledge about the results.” For the question regarding “satisfaction with the time consumed for completing disease screening,” the average rating was 4.08. Three patients chose “neutral,” five chose “satisfied,” and four chose “very satisfied.” The five comments received in response to this question all emphasized the speed of the process, with phrases such as “Quick” or “Fast.” All 12 respondents rated their “likelihood of recommending the facility” (question 4) as 4 or higher, resulting in an average rating of 4.33. Only two participants provided comments regarding their impressions of the service. Both expressed a positive sentiment and referred to the intervention as “innovative.” The low number of patients who filled out the questionnaire can be attributed to fatigue after spending a number of hours in the clinic.

## Discussion

In Germany, the scope of patient screening for chronic eye disease within the general health insurance system is currently limited to screening for DR in patients with confirmed diabetes mellitus. This process requires a formal referral from a general practitioner to an ophthalmologist, who then carries out the examination. Despite these measures, it is noteworthy that only an average of 50% of diabetic patients adhere to the recommended ophthalmologist visits [35]. The screening for diabetic retinopathy is a solitary process, with no other healthcare professionals involved. The role of fundus photography grading, whether by professional graders or AI, is yet to be recognized in the German healthcare system [36, 37].

This study established a clinical setting wherein a healthcare provider could perform chronic eye disease screening using a combination of hardware and software solution. Additionally, the study explored methods for enabling remote screening audits involving specialists. To the best of our knowledge, this study represents one of the pioneering attempts to incorporate screening for diseases DR, AMD, and glaucoma within a single system.

In terms of the measured data, the DR model demonstrated remarkable performance by accurately detecting all patients with DR disease, achieving a sensitivity of 100%. Out of the 29 gradable AMD patients, the AMD model successfully identified 26 patients. The remaining three patients were not detected, including one with “RPE Defects of the macula,” one with “choroidal nevus located superior to the optic nerve” and one patient with AMD in one eye. As for glaucoma grading, excluding the ungradable patients, a total of 85 patients were classified as “suspect glaucoma” and required referral according to the auditor’s assessment. The glaucoma model accurately identified 78 out of these 85 patients.

The specificities of DR, AMD, and glaucoma gradings appear to be relatively low compared to their sensitivities. However, upon closer investigation, several phenomena and facts emerge to explain these observations.

Regarding DR, among the false positive gradings, it was observed that 90% of patients required “ophthalmologist referral” according to the auditor. This suggests that the DR model detected certain fundus images with abnormalities resembling DR lesions or patterns similar to those, for instance, ten patients were identified with “referable AMD”. The others present with abnormalities such as “peripheral bleeding” and “laser scars”, etc. Collectively, these various abnormalities contributed to a final false referral rate of only 10% among the false positive DR patients.

In the case of AMD, the false positive gradings exhibited a similar pattern to that of the false positive DR gradings. Among these, 88% of the patients required an “ophthalmologist Referral”. This indicates that the AMD model identified certain eye images displaying abnormalities that resembled AMD lesions or patterns. Interestingly, seven patients were classified as “referable” DR patients, suggesting that the presence of DR features influenced the AMD model’s grading. Additionally, other abnormalities such as “retinal scars"and “epiretinal membrane”, etc.were observed. These various abnormalities contributed to a final false referral rate of only 12%.

In the context of Glaucoma, even among the false positive gradings, a substantial number (63.65%) necessitated an “ophthalmologist referral” In the remaining cases (12 patients), no additional abnormalities were detected, except for one patient who exhibited “minor RPE defects”.

Furthermore, when combining all false positives (84 patients),, we found that only 17 patients (20%) needed to follow the annual screening. All the remaining 67 patients needed to be referred to ophthalmologists within a timeframe ranging from one week to three months. This analysis suggests that the AI system did not significantly increase the rate of incorrect referrals in the clinical trial for the three-disease screening.

Based on the analysis conducted, it is evident that the misgrading of DR and AMD, leading to false positives, was primarily due to the presence of other abnormalities that the models might not have been trained to differentiate as distinct diseases. Additionally, we observed that certain images displaying AMD or DR features influenced the accurate classification of the two diseases. In the case of glaucoma, as it relies solely on the image data around the disc region without considering measures such as intraocular pressure (IOP) and visual field, its sensitivity and specificity are relatively low.

Table 7 presents a comparative analysis of the grading performance of the systems introduced in the backgroundsection of this paper. It is worth mentioning that RAIDS not only serves the same function as our system in grading three diseases but also extends its capabilities to identify other eye abnormalities. In contrast, the trials conducted for the other systems were exclusively centred on grading DR.

**Table 7:** Comparison of the performance of referable DR grading from different systems in clinic settings

IDx-DR (DR)	EyeArt (DR)	SELENA+ (DR)	Offline AI System* (DR)	VoxelCloud Retina (DR)	RAIDS (DR, AMD, and Glaucoma)	TeleEye MD (DR, AMD, and Glaucoma)
Validated on 900 patients SE: 87.2%; SP: 90.7% IDx-DR 2.0 Validated on 1415 patients in the Hoorn Diabetes Care System SE: 91% SP: 84% (Using EURODIAB criteria)	EyeArt v2.1 Validated on 30,405 patients in the English Diabetic Eye Screening Programme in the UK SE: 95.7% SP: 54.0% Validated on 893 patients SE: 95.5% SP: 85.3%	Validated on 1574 patients in a mobile screening program in Zambia SE: 92.3% SP: 89.0% Validated on 69 patients in Australia SE: 96.9% SP: 87.7%	Validated on 236 participants in Australia SE: 96.9% SP: 87.7%	Validated on 15,805 patients at 155 diabetes centres in China SE: 83.3% SP: 92.5%	Validated on 110,784 participants from 65 healthcare centers in China DR SE: 83.7% SP: 98.6% AMD SE: 88.1% SP: 97.6% Referral possible glaucoma SE: 81.3% SP: 95.0%	Validated on 231 Patient in this study DR SE: 100.0% SP: 80.1% AMD SE: 90.9% SP: 78.8% Referral possible glaucoma SE: 93.2% SP: 76.8%

* From the Centre for Eye Research, Australia

As previously mentioned, the factors related to human-computer interaction can influence the performance of AI applications in real-world clinical settings [27]. In our study, we conducted preliminary investigations into these factors. We observed that only 5 out of the patients with both eyes were deemed ungradable. One contributing factor to this limited ungradability could be attributed to the well-established imaging room setup within a hospital environment and the generally high image quality produced by the fundus camera. Regarding the user experience of clinical staff, the feedback from the study nurse indicated that the patient registration device and the fundus camera were user-friendly and easy to operate for patient information input and image capture. Considering the seamless operation of the camera and the bridging device, we did not observe any substantial influence from other factors such as data transmission speed, the image acquisition and analysis workflow on the disease grading performance. The system received positive feedback from the medical staff involved in the auditing process. All these underscore the significance of a well-designed human-computer interaction in clinical AI applications, as it can enhance both user experience and diagnostic accuracy.

However, we observed and experienced several limitations that must be taken into account when interpreting our results. Firstly, the sample size of our study was small, which may impact the accuracy of the three AI models we tested. Additionally, the ethnic background of our participants was limited to Caucasians only. We used only one camera in our study. Furthermore, we only collected feedback from a limited number of participants, with only 12 forms collected. The grading and severity of diseases we assessed were based solely on the colour fundus images, and other imaging modalities or measurements, such as OCT or IOP, were not utilized to determine the ground truth. Looking forward, the development and implementation of operator-independent methods show promise. This includes the further exploration of smartphone-based visual field measurement tools and the utilization of virtual reality headsets, both of which hold potential for enhancing glaucoma screening in primary care settings. We hope that future studies can build upon our findings to address these limitations and further advance this field.

## Conclusion

The implementation of the AI-based approach for screening three chronic eye diseases has demonstrated its effectiveness in real-world settings, especially when comparing the individual disease’s referral decisions and their combined referral rate. Both the screening staff and auditor have expressed positive feedback regarding the ease of use of the hardware and software platform. The incorporation of an auditing function has proven valuable for obtaining timely second opinions from experts, potentially applicable for facilitating remote screening.

The detection of multiple eye diseases carries significant importance due to their potential to cause visual impairment, coupled with their increasing prevalence. Considering the ongoing global advancements in technology and the evolving demographic landscape in Germany, the integration of AI into disease screening approaches emerges as a logical and necessary progression. Continued research and development in this field will further refine the accuracy and effectiveness of AI systems, ultimately benefiting individuals affected by these chronic eye diseases.

## Data Availability

The datasets generated and/or analysed during the current study are not publicly available due to patient data protection but are available from the corresponding author on reasonable request.

## Abbreviations

AI: Artificial intelligence
AMD: Age-related macular degeneration
AUC: Area under the receiver operating characteristics curve
DME: Diabetic macular edema
DR: Diabetic retinopathy
IOP: Intra-Ocular Pressure
IQR: Interquartile range
mtmDR: More than mild diabetic retinopathy
NPDR: Non proliferative diabetic retinopathy
NPV: Negative predictive value
OCT: Optical coherence tomography
PDRP: Proliferative diabetic retinopathy
PPQ: Positive predictive value
QA: Quality assessment
VTDR: Vision-threatening diabetic retinopathy
